# Exploring the respiratory viral landscape beyond influenza and SARS-CoV-2: a 2020–2023 study in Fujian, China

**DOI:** 10.3389/fpubh.2025.1558716

**Published:** 2025-05-14

**Authors:** Libin You, Zhengtao Zhang, Hongjin Li, Yuwei Weng

**Affiliations:** ^1^Key Laboratory of Fujian Province for Zoonotic Disease Research, Fujian Center for Disease Control and Prevention, Fuzhou, China; ^2^Nanping First Hospital, Nanping, China

**Keywords:** acute respiratory infection, common respiratory viruses, prevalence, influenza-negative, SARS-CoV-2

## Abstract

**Introduction:**

In this study, we investigated the local epidemiology and pathogen spectrum of acute respiratory infections caused by common respiratory viruses other than influenza virus (IFV) and severe acute respiratory syndrome coronavirus 2 (SARS-CoV-2) in Fujian Province, China, from September 2020 to December 2023.

**Methods:**

Samples negative for IFV and SARS-CoV-2 were randomly selected from individuals presenting with influenza-like illness. These samples were tested for seven common respiratory viruses—human respiratory syncytial virus (HRSV), human parainfluenza virus (HPIV), human adenovirus (HAdV), rhinovirus (RV), human metapneumovirus, human coronavirus (HCoV), and human bocavirus. Quantification fluorescence polymerase chain reaction (qPCR) was used for detection.

**Results:**

One or more respiratory viruses were identified in 30.2% (*n* = 1,010/3,345) of the collected samples. RV was the most prevalent (10.9%), followed by HPIV (6.2%) and HRSV (4.5%). HPIV-3 was the dominant HPIV subtype (44.0%), and HCoV-OC43 was the predominant HCoV genotype (54.4%). Co-infections were observed in 2.9% (*n* = 96) of cases, with RV and HPIV co-infection being the most frequent. Age-specific variations were observed for most viruses, except for HCoV. HRSV exhibited a notably higher prevalence in young children (<5 years) and seniors (≥60 years), while HAdV was more common in children younger than 15 years. Regarding seasonal distribution, RV peaked in spring and autumn, whereas HRSV peaked in summer and autumn. No clear seasonal trends were observed for HPIV and HAdV. A negative correlation was observed between the incidence of IFV and the seven targeted respiratory viruses.

**Discussion:**

These findings collectively underscore the importance of age-specific and seasonally tailored interventions, as well as the need for comprehensive diagnostic tools capable of simultaneously identifying multiple pathogens.

## 1 Introduction

Acute respiratory infection (ARI) is one of the most common infectious diseases globally, causing significant human morbidity and mortality (1). In 2019, pneumonia and other lower respiratory infections constituted the deadliest group of communicable diseases worldwide, collectively ranking as the fourth leading cause of death (2). ARIs can result in mortality across all age groups. Current evidence suggests that ARI is the leading cause of infectious disease-related deaths in children under 5 years of age, accounting for 15% of total deaths in this age group, with a global average mortality rate as high as 118.9 per 100,000 (3).

ARIs typically present as polymicrobial infections involving multiple pathogenic microorganisms. In recent years, the etiological profile of community-acquired pneumonia (CAP) has undergone significant changes, with a notable increase in the detection rate of viral pneumonia and a corresponding decrease in bacterial pneumonia (4–6). A wide range of human respiratory viruses, including influenza virus (IFV), severe acute respiratory syndrome coronavirus 2 (SARS-CoV-2), human respiratory syncytial virus (HRSV), human parainfluenza virus (HPIV), human adenovirus (HAdV), rhinovirus (RV), and human coronavirus (HCoV), can cause ARIs. Due to geographical, climatic, and other factors, the prevalence and spectrum of pathogens causing ARIs exhibit substantial heterogeneity across different countries and regions. Situated in the subtropical southeastern coastal zone of China, Fujian Province experiences a warm and humid climate. With its densely populated areas and pronounced seasonal climate shifts, the region is predisposed to recurrent ARI outbreaks caused by various respiratory pathogens. Therefore, understanding the local epidemiological landscape, pathogen spectrum, and influencing factors of ARIs is crucial for formulating comprehensive prevention and control strategies tailored to local conditions, as well as optimizing the allocation and utilization of medical and healthcare resources.

Prior to 2020, etiological surveillance of ARIs in China primarily focused on the influenza virus. Following the 2019 coronavirus disease (COVID-19) pandemic, SARS-CoV-2 was included in systematic surveillance. However, the potential impact of other respiratory viruses on ARI epidemics remained largely uncharacterized. Data from the China National Influenza Center revealed that the mean nucleic acid testing positive rate for IFV among influenza-like illness (ILI) cases in Fujian Province between 2015 and 2019 was only 14.7%. This finding suggested that a substantial proportion of ARI cases, potentially exceeding 80%, could be attributed to pathogens other than IFV. It is widely recognized that the burden of non-influenza ARIs is significant. Despite intensified surveillance efforts for SARS-CoV-2 and influenza virus during the COVID-19 pandemic, the epidemiological and etiological characteristics of ARIs caused by other respiratory viruses in Fujian Province remained poorly understood. To address this knowledge gap, we conducted a prospective study. ILI samples were collected in Fujian Province, excluding those that tested positive for IFV and SARS-CoV-2. These samples underwent comprehensive screening using quantification fluorescence polymerase chain reaction (qPCR) detection methods. The primary objective of this study was to elucidate the spectrum and distribution of pathogens in ARI cases caused by various viruses other than IFV and SARS-CoV-2 in Fujian Province.

## 2 Materials and methods

### 2.1 Ethics statement

The samples utilized in this study were obtained from the intensified surveillance program for SARS-CoV-2 and influenza virus. No additional sampling was conducted for this specific study. All samples were uniformly renumbered to ensure complete de-identification and the removal of any personal information. As a result, ethical approval for this study was not deemed necessary.

### 2.2 Patients and sample collection

All samples in this study were collected from sentinel hospitals participating in ILI surveillance across nine prefecture-level cities in Fujian Province. Each week, 20 oropharyngeal swabs were obtained from ILI cases, defined as individuals presenting with a fever of 38°C or higher and exhibiting respiratory symptoms such as sore throat and/or cough, at each sentinel hospital. These samples were subsequently transported to the respective Municipal Centers for Disease Control and Prevention (CDC) for nucleic acid testing of influenza virus and SARS-CoV-2. Among the samples that tested negative for both influenza virus and SARS-CoV-2, approximately 10 samples per month were randomly selected from each municipal CDC for further screening of multiple viral pathogens. To ensure both randomness and representativeness in the selection process, a systematic sampling method was employed. The negative samples were sorted by their unique identification numbers, and every fifth sample in the sequence was chosen for analysis. This method resulted in the submission of approximately 10 samples per month to our laboratory for further examination. The identification numbers were randomly generated, minimizing the risk of periodic bias. All samples were stored at −80°C immediately after collection and tested within 2 months.

Between September 2020 and December 2023, a total of 3,345 oropharyngeal swab samples were collected, yielding a mean of 84 samples per month (range: 68–97). The study population consisted of 1,818 males and 1,527 females, resulting in a male-to-female ratio of 1.19. Patient ages ranged from approximately 1 month to 94 years, with a median age of 9 years (inter-quartile range [IQR]: 3–30 years). For comparative analysis, participants were stratified into five age groups: young children (0–4 years), children (5–14 years), adolescents and young adults (15–24 years), adults (25–59 years), and older adults (≥60 years).

### 2.3 DNA/RNA extraction

Viral genomic DNA or RNA was extracted using a Viral Nucleic Acid Isolation Kit (TIANLONG, Xi’an, China) in conjunction with the GeneRotex System (TIANLONG, Xi’an, China), following the manufacturer’s instructions.

### 2.4 Multiplex real-time qPCR detection

The qPCR screening was performed using the Multiplex Real-Time PCR Diagnostic Kit (D334LYH, Abtechnology, Beijing, China). This kit is specifically designed for the detection of various respiratory pathogens. The kit comprises four distinct multiplex reactions that collectively enable the identification of six species of respiratory viruses: HRSV, HPIV, human metapneumovirus (HMPV), HCoV, RV, and human bocavirus (HBoV). Furthermore, the kit can differentiate among the four serotypes of HPIV (HPIV-1, −2, −3, −4) and four genotypes of HCoV (HCoV-229E, -OC43, -HKU1, -NL63). qPCR assays were performed on an ABI QuantStudio 7 Dx real-time PCR system (Thermo Fisher Scientific, Massachusetts, United States) following the manufacturer’s instructions.

### 2.5 Human adenovirus detection

Primers and probes specific for adenovirus genogroups B, C, and E associated with ARIs were designed and selected based on the Penton gene sequence of HAdV-2 (accession number: AC_000007). Primer-Premier 5 (Premier Biosoft International, Palo Alto, CA) and MAFFT (7) were utilized for primer design. The forward primer (5’-ACCATYACCACCGTCAGTGA-3′), reverse primer (5’-CGGTAG SGTCCCGTGATCT-3′), and probe (5’-FAM-AACGTTCCTGCT CTC-TAMRA-3′) were synthesized by Sangon Biotech Co., Ltd., Shanghai, China.

One-step real-time PCR was performed to detect HAdV using the Path-ID™ qPCR Master Mix (Applied Biosystems) and the primers/probes specified above. The qPCR reaction was carried out in a total volume of 25 μL, comprising 12.5 μL of 2 × PCR master mix (containing an optimized buffer system, DNA polymerase, MgSO4, and dNTPs), 2 μL of primers, 0.5 μL of probe (each at a concentration of 10 μmol/L), 5 μL of nuclease-free water, and 5 μL of template DNA. Thermal cycling was performed using the CFX-96 Real-Time System (Bio-Rad, Hercules, United States), with an initial denaturation step at 95°C for 10 min, followed by 40 cycles of denaturation at 95°C for 15 s and annealing/extension at 60°C for 1 min. The threshold cycle (Ct) value was defined as the cycle number at which the fluorescence signal crossed the background threshold. A sample was considered positive if it exhibited a complete amplification curve and yielded a Ct value ≤ 38, indicating the presence of detectable viral DNA.

### 2.6 Influenza and SARS-CoV-2 surveillance data collection

To investigate the interrelationships between other respiratory viral infections and the prevalence of influenza virus and SARS-CoV-2, we conducted a comprehensive data acquisition process using the China Information System for Disease Control and Prevention. Our data collection specifically focused on the number of influenza virus tests performed and the corresponding number of influenza virus-positive cases throughout the study period. The monthly positive rate for influenza virus was calculated by dividing the number of influenza virus-positive samples by the total number of tests conducted in each month. Monthly figures for confirmed COVID-19 cases in Fujian Province were obtained from September 2020 to December 2023.

### 2.7 Statistical analysis

Statistical analyses were performed using SPSS version 23.0 (SPSS Inc., Chicago, IL, United States). Descriptive statistics were calculated for all variables. Chi-square (χ^2^) tests were used to assess differences in viral positivity rates across age, gender, year, and season. Statistical significance was set at *p* < 0.05.

The correlation between the positive detection rates of seven targeted respiratory viruses and the influenza virus was assessed using Spearman’s rank correlation coefficient. Two-sided *p*-values less than 0.05 were considered statistically significant.

## 3 Results

### 3.1 Detection of respiratory viruses

A total of 1,010 of 3,345 samples (30.2%) tested positive for one or more respiratory viruses. Of these, 914 samples (27.3%) were positive for only one of the 13 viruses examined, which included four serotypes of HPIV and four genotypes of HCoV. Additionally, 89 samples (2.7%) were positive for two viruses, and 7 samples (0.2%) were positive for three viruses.

**Figure 1:**
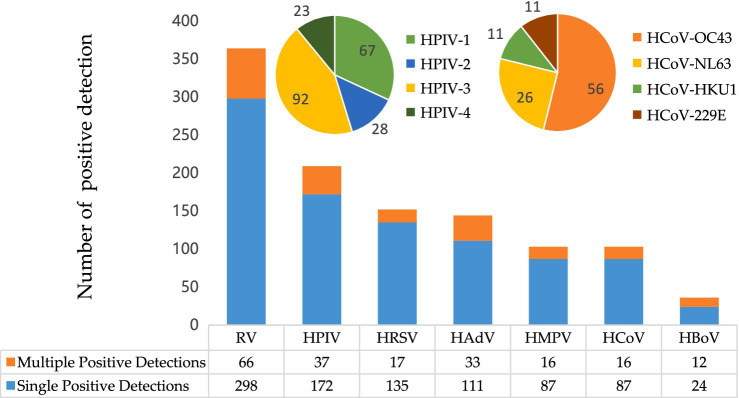
Prevalence of respiratory virus detections. RV, rhinovirus; HPIV, human parainfluenza virus-1, −2, −3, −4; HRSV, human respiratory syncytial virus; HAdV, human adenovirus; HMPV, human metapneumovirus; HCoV, human coronavirus-229E, -OC43, -HKU1 -NL63; HBoV, human bocavirus.

Among the seven common respiratory viruses detected, RV was the most prevalent (10.9%, 364/3,345), followed by HPIV (6.2%, 209/3,345) and HRSV (4.5%, 152/3,345; Figure 1). Among the HPIV-positive samples, HPIV-3 was the most common (44.0%, 92/209), followed by HPIV-1 (32.1%, 67/209), HPIV-2 (13.4%, 28/209), and HPIV-4 (11.0%, 23/209). Among the HCoV positive samples, HCoV-OC43 was the predominant genotype (54.4%, 56/103). The remaining three HCoV genotypes were detected less frequently: HCoV-NL63 (25.2%, 26/103), HCoV-229E (10.7%, 11/103), and HCoV-HKU1 (10.7%, 11/103).

Multiple viral detections (samples testing positive for more than one virus) were observed in 96 of 3,345 samples (2.9%). RV was the most frequently identified virus in these multiple detections (68.8%, 66/96) and co-occurred with all other respiratory viruses detected in this study. Of the multiple positive detections, the co-existence of RV and HPIV was the most common (26/96), followed by the co-existence of RV and HAdV (22/96).

### 3.2 Demographic characteristics

**Figure 2:**
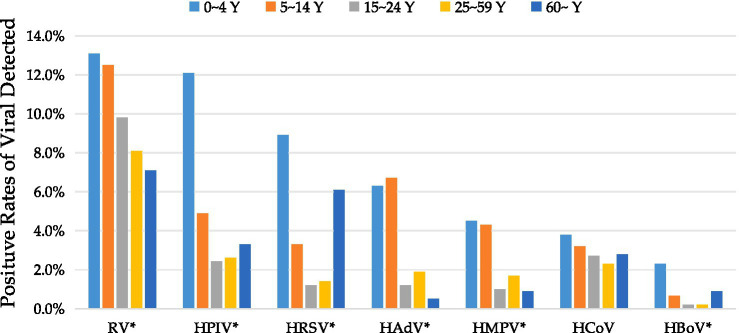
Positivity rates of different respiratory viruses across various age groups. *The asterisk denotes a significant difference in positivity rates between age groups (*p* < 0.05). Y, years.

Of the 1,010 positive samples, 564 (55.8%) were from males and 446 (44.2%) from females. There was no significant difference in positivity rates between the genders (χ^2^ = 1.298, *p* = 0.255). The overall positivity rate of the seven targeted respiratory viruses differed significantly among age groups (*p* < 0.01; Supplementary Table S1). Children under 5 years of age presented with the highest positivity rate (45.1%, 483/1,072), while individuals aged 25 years and older had the lowest (17.2%, 145/843; Supplementary Table S1). Figure 2 shows the prevalence of individual respiratory viruses by age group, demonstrating a concentration of positive detections in children under 5 years old. Significant differences in positivity rates were observed across age groups for several respiratory viruses (*p* < 0.01), except for HCoV (*p* > 0.05).

The positivity rate of RV decreased with increasing age. However, the positivity rates of other viruses showed a decreasing trend only in the first three age groups, except for HAdV, which peaked in the 5–10 year age group.

### 3.3 Seasonal fluctuations in respiratory virus prevalence

From 2021 to 2023, the annual viral positivity rates were 29.0% (279/961), 33.3% (344/1,032), and 25.5% (259/1,015), respectively. Analysis of the annual positivity rate excluded the year 2020, as data were only available from September to December of that year. The difference in viral detection rates across these years was statistically significant (χ^2^ = 25.672, *p* < 0.01). Seasonal comparisons of respiratory virus positivity rates among ARI patients are detailed in Table 1. The positivity rates for all seven respiratory viruses during spring (March through May), summer (June through August), autumn (September through November), and winter (December through February) were 29.8% (225/756), 31.2% (239/767), 34.3% (333/971), and 25.0% (213/851), respectively. These seasonal differences were statistically significant (*p* < 0.01). Notably, the positivity rate of RV was significantly higher in spring and autumn (*p* < 0.05). HRSV showed a markedly higher positivity rate during summer and autumn (*p* < 0.05). In contrast, the positivity rates for HPIV and HAdV remained relatively stable throughout the year, with no clear seasonal trends (*p* > 0.05). The other viruses—HMPV, HCoV and HBoV—presented relatively low positive rates year-round, without any pronounced seasonal variations (*p* > 0.05).

**Table 1:** Seasonal comparison of the incidence of other respiratory virus infections during the study period [n(%)].

Pathogen	Spring (*N* = 756)	Summer (*N* = 767)	Autumn (*N* = 971)	Winter (*N* = 851)	*χ* ^2^	*p*
Total	225 (29.8)	239 (31.2)	333 (34.3)	213 (25.0)	18.922	0.000
RV	111 (14.7)	67 (8.7)	123 (12.7)	63 (7.4)	28.717	0.000
HPIV	42 (5.6)	41 (5.3)	72 (7.4)	54 (6.3)	6.056	0.417
HRSV	19 (2.5)	60 (7.8)	57 (5.9)	16 (1.9)	44.056	0.000
HAdV	36 (4.8)	27 (3.5)	40 (4.1)	41 (4.8)	2.154	0.541
HMPV	20 (2.6)	22 (2.9)	24 (2.5)	37 (4.3)	6.381	0.094
HCoV	15 (2.0)	30 (3.9)	37 (3.8)	21 (2.5)	10.544	0.104
HBoV	4 (0.5)	10 (1.3)	13 (1.3)	9 (1.1)	3.130	0.372

### 3.4 Association with influenza virus infection and COVID-19 pandemic

**Figure 3:**
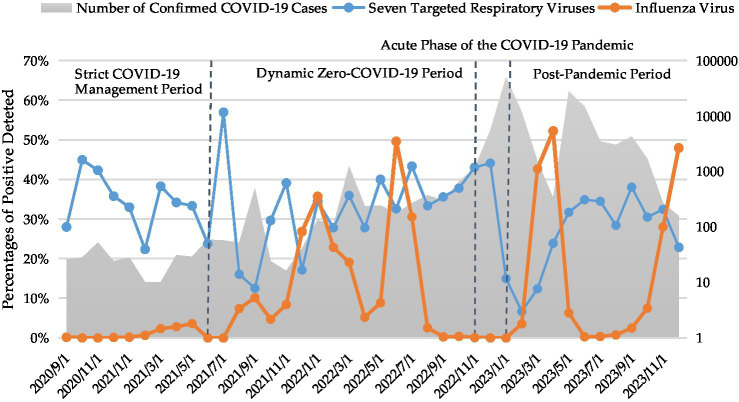
Monthly number of confirmed COVID-19 cases and percentages of positive detections of influenza virus and seven targeted respiratory viruses.

Statistical analysis of the relationship between influenza incidence and the detection rates of seven targeted respiratory viruses revealed a significant negative correlation (Spearman’s correlation coefficient = −0.325, *p* < 0.05). This inverse association indicated that an increase in influenza incidence tended to be accompanied by a decrease in the detection rates of these respiratory viruses, and vice versa (Figure 3).

Given the study period encompassed the COVID-19 pandemic, we categorized the research timeline into four distinct phases based on evolving epidemic prevention and control policies in Fujian Province. These phases include: (1) the Strict COVID-19 Management Period (September 2020–June 2021), during which no local transmissions occurred, but imported cases were present, and stringent control measures were implemented; (2) the Dynamic Zero-COVID-19 Period (July 2021–November 2022), initiated with the release of the ninth version of the national prevention and control plan; (3) the Acute Phase of the COVID-19 Pandemic (November 2022–January 2023), a critical transition period marked by a shift from stringent measures to “Category B management with Precautions,” culminating in a peak of cases on December 22; and (4) the Post-Pandemic Period (February–December 2023).

As presented in Table 2, following the implementation of the COVID-19 Class-B management with optimized control protocols, a significant decline in the overall positivity rate of the seven targeted respiratory viruses was observed from the acute phase of the COVID-19 pandemic to the post-pandemic period (*p* < 0.05). Upon further analysis of individual pathogens, the positivity rates of HRSV, RV, HAdV, and HCoV did not exhibit significant differences across the four defined stages. Conversely, significant differences in positivity rates were observed among the four stages for HMPV, HBoV, and HPIV.

**Table 2:** Positivity rates of respiratory virus across different epidemic phases [n(%)].

Pathogen	Phase I (*N* = 846)	Phase II (*N* = 1,416)	Phase III (*N* = 155)	Phase IV (*N* = 928)	*χ* ^2^	*p*
Total	284 (33.6)	472 (33.3)	43 (27.7)	249 (26.8)	14.269	0.003
RV	106 (12.5)	155 (10.9)	12 (7.7)	91 (9.8)	5.058	0.168
HPIV	71 (8.4)	91 (6.4)	6 (3.9)	41 (4.4)	15.858	0.015
HRSV	30 (3.5)	73 (5.2)	3 (1.9)	46 (5.0)	5.959	0.114
HAdV	37 (4.4)	58 (4.1)	8 (5.2)	41 (4.4)	0.464	0.927
HMPV	38 (4.5)	32 (2.3)	3 (1.9)	30 (3.2)	9.594	0.022
HCoV	29 (3.4)	47 (3.3)	6 (3.9)	21 (2.2)	6.210	0.400
HBoV	4 (0.5)	22 (1.6)	4 (2.6)	6 (0.6)	10.829	0.013

Phase I: strict COVID-19 management period (Sept 2020–Jun 2021); Phase II: dynamic zero-COVID-19 period (Jul 2021–Nov 2022); Phase III: acute phase of COVID-19 (Dec 2022–Jan 2023); Phase IV: post-pandemic period (Feb–Dec 2023).

## 4 Discussion

In China, the prevalence of ARIs exhibits regional variability due to diverse geographical, environmental, and climatic factors. Continuous surveillance of pathogen composition and ARI prevalence is crucial for effective prevention and control of respiratory infectious diseases. This study comprehensively analyzed common respiratory viral infections from 2020 to 2023, providing valuable insights into the epidemiological landscape of non-influenza and non-SARS-CoV-2 respiratory viruses within Fujian Province, China.

The study period exhibited substantial fluctuations in virus detection rates, with marked interannual variations. Comparable annual oscillations were observed in other studies (8). This variability can be ascribed to a confluence of factors, including shifts in population immunity (9), human mobility patterns (10), and the impact of non-pharmaceutical interventions on community transmission (11) during the COVID-19 pandemic.

The detection of respiratory viruses other than influenza virus and SARS-CoV-2 in approximately one-third of the samples (30.2%) underscored their substantial contribution to ARIs. The present study revealed a pronounced age-related pattern of viral infection, with children under 5 years of age comprising 47.8% (483/1,010) of positive samples. This high prevalence in young children was likely attributable to their developing immune systems and frequent exposure within communal settings such as childcare centers. Children aged 5 to 14 years also exhibited a considerable positive detection rate (33.0%), emphasizing the importance of targeted interventions within educational and social institutions frequented by children to minimize transmission.

The predominance of RV as the foremost viral pathogen (10.9%) aligned with findings from a previous study conducted in Guangdong Province between 2019 and 2021 (12). This contrasts with the predominance of HRSV observed in several studies conducted before the COVID-19 pandemic (13–15). While IFV was prevalent, an epidemiological investigation conducted in Fuzhou in 2019 identified HAdV as the leading respiratory pathogen of ARI (16).

Among the commonly investigated viruses, HPIV demonstrated the second highest prevalence (6.2%), with HPIV-3 as the predominant subtype (44.0% of HPIV-positive detections). This finding aligned with pre-COVID-19 studies conducted in Chengdu, China (17). A study from South Africa observed a shift in dominant serotype of HPIV during the pandemic, with subsequent waves primarily driven by HPIV-3 while circulation of HPIV-1, −2, and −4 declined (18). Similarly, a delayed HPIV-3 outbreak peaked in Gwangju, South Korea, in 2021 (19). Genetic analysis revealed reduced diversity among HPIV-3 strains, raising concerns that the emergence of new lineages could compromise existing collective immunity. The high incidence of HPIV necessitates further investigation and the implementation of potential surveillance measures.

In the present study, HRSV was detected in 4.5% of samples, with a predominance of positive cases observed in young children and the older adults. Prior research has extensively documented the substantial health burden imposed by HRSV, particularly within these vulnerable populations, including infants, the older adults, and immunocompromised individuals, leading to severe respiratory illnesses (20, 21). Our findings corroborate the heightened susceptibility of these age groups to HRSV infections, underscoring the critical need for the implementation of targeted preventive strategies, such as vaccinations, and the provision of specialized healthcare services tailored to their unique requirements.

It is generally acknowledged that most respiratory virus infections exhibit seasonality. In temperate regions, these viruses typically demonstrate higher prevalence during winter, while they peak during the rainy season in tropical areas (22). Previous studies have established that outbreaks of influenza virus, HRSV, and HCoV commonly occur during the winter season, while other human respiratory viruses, such as HPIV, HMPV and RV showed highest activity during the spring or autumn seasons in temperate climates (23). This study preliminarily observed distinct seasonal patterns in the prevalence of certain respiratory viruses in Fujian Province, a region with a subtropical climate. RV demonstrated increased activity during spring and autumn, while HRSV displayed higher detection rates during summer and autumn. HPIV exhibited steady epidemic intensity throughout the year without apparent seasonal trends, and the other respiratory viruses appeared to occur sporadically year-round.

The simultaneous detection of multiple viruses within a single patient sample indicates co-infection with several respiratory pathogens. The identification of co-infections in 2.9% of patients in this study highlights the intricate nature of respiratory viral infections and emphasizes the critical need for comprehensive diagnostic testing. Among these co-infections, RV emerged as the most prevalent pathogen, exhibiting a notable association with HPIV. Notably, previous studies have documented severe clinical outcomes associated with RV co-infections, including a reported case of severe pneumonia with RV/HBoV co-infection (24) and another involving a 3-month-old infant who succumbed to acute respiratory distress syndrome and multi-organ thrombotic microangiopathy due to RV/HCoV-229E co-infection (25). Amat et al. (26) suggested RV/HRSV co-infection was a risk factor for recurrent bronchial obstruction. Nevertheless, the clinical impact of co-infections involving RV and other respiratory viruses, such as synergistic or antagonistic effects, remains less understood. Whether RV co-infection would result in more severe clinical outcomes remains inconclusive among different studies (27, 28). In our study, the observed predominance of co-infections in children under 5 years of age underscores their heightened susceptibility to these infections, necessitating the implementation of tailored clinical management and robust infection control measures.

The multifaceted strategies implemented to mitigate the transmission of SARS-CoV-2 likely had a significant impact on the circulation of other respiratory pathogens. Epidemiological and clinical characteristics of ARIs notably shifted during the COVID-19 pandemic (29). Kurskaya et al. (30) documented dramatic fluctuations in the prevalence of common respiratory viruses before and during the COVID-19 pandemic. Fernanda et al. (8) even found an absence of detection of HRSV and IFV during the pandemic in a Brazilian cohort. In Fujian, China, distinct phases of COVID-19 prevention and control were observed. Strict isolation policies during initial phases effectively suppressed local transmission, followed by a period of more relaxed measures leading to sporadic outbreaks. The recent shift toward Class B management policies has resulted in a rapid surge in infections, transitioning into a post-epidemic phase. An analysis of pathogen epidemic patterns across distinct phases of the COVID-19 outbreak demonstrated a significant decrease in the overall positivity rate of seven targeted respiratory viruses during both the acute and post-pandemic periods compared to earlier stages. While stringent quarantine measures effectively suppressed the transmission of SARS-CoV-2, they did not substantially diminish the prevalence of other respiratory viruses within the population. This finding underscores the critical role of efficient isolation strategies in preventing and controlling the transmission of respiratory viruses among individuals. Conversely, the data suggest that personal protective measures such as mask-wearing and frequent hand hygiene may have a relatively limited impact on the broader spectrum of respiratory virus circulation.

Further examination of pathogens revealed no significant variation in positivity rates for HRSV, RV, HAdV, and HCoV across all four epidemic stages in this study. Conversely, positivity rates for HPIV and HMPV gradually declined as epidemic intensity increased but rebounded after the conclusion of the epidemic period. Notably, the positivity rate of HBoV increased concurrently with the rise in COVID-19 cases and subsequently decreased to a low prevalence level in the post-pandemic period. Several studies have also demonstrated that the clinical manifestation of RV remained unchanged, establishing it as a predominant co-circulating virus during the COVID-19 pandemic (8, 27, 29, 31). This phenomenon can be attributed to RV’s diverse modes of transmission and the absence of an enveloped structure, rendering it more resistant to common disinfectants and soaps, thereby contributing to its continued prevalence (32). These findings indicate that future public health initiatives should adopt tailored intervention strategies based on the specific characteristics of viral agents. For instance, emphasis should be placed on enhancing environmental elimination measures for RV, rather than relying exclusively on social isolation.

The observed negative correlation between influenza and other respiratory viruses suggests potential competition among these pathogens within the human population. The dominance of influenza virus may suppress the circulation of other respiratory viruses. This phenomenon could be attributed to various factors, including viral interference, altered human behavior in response to influenza outbreaks, or shifts in host immunity that favor influenza over other viruses (33). Anestad et al. (34) hypothesized that influenza virus infection might confer short-lived, non-specific immune protection, potentially decreasing susceptibility to other respiratory viral infections. Several studies have also demonstrated that influenza virus infection reduced the risk of non-influenza respiratory infections, or that the circulation of other respiratory viruses was delayed during influenza seasons (33, 35, 36). This phenomenon has significant implications for the control of ARIs, highlighting the need for surveillance strategies that screen for a broader range of viruses beyond influenza virus. Further research is crucial to elucidate the underlying mechanisms of this phenomenon and to assess the effectiveness of current respiratory virus control and prevention efforts.

This study has several limitations that should be acknowledged. Firstly, the investigation was restricted to a specific set of respiratory viruses, excluding other potential pathogens such as bacteria and mycoplasma. This may have led to an underestimation of the diversity of respiratory pathogens contributing to ARIs. Secondly, as the study focused on samples negative for both influenza and SARS-CoV-2, the findings may not accurately reflect the overall prevalence of ARIs within the general population. However, the results provide valuable insights into the infection status of seven common respiratory viruses within the population of Fujian province.

In summary, this study expanded our understanding of the epidemiology of common respiratory viruses beyond influenza and SARS-CoV-2. Key findings underscored the significance of age-specific and seasonally-tailored interventions. Based on the observed age and seasonal distribution patterns, we propose actionable strategies, such as implementing disinfection protocols in schools and prioritizing multiplex testing for children during peak RV activity in spring and autumn to address wheezing cases. Besides, enhancing early detection and supportive care for the older adults during HRSV surges in summer and autumn can be achieved through community-based rapid testing networks. Another promising avenue for future research and public health planning involves leveraging seasonal data to develop predictive models that optimize healthcare resource allocation in the Fujian Province. Our analysis of viral circulation patterns across different phases of COVID-19 further highlights the need for public health strategies to adapt to the unique transmission dynamics of specific pathogens. Continued surveillance remains essential, particularly for vulnerable populations like young children and the older adults, remains critical to improving responsiveness to emerging respiratory threats.

## Data Availability

The raw data supporting the conclusions of this article will be made available by the authors, without undue reservation.

## References

[1] Moesker FM van Kampen JJ van Rossum AM de Hoog M Koopmans MP Osterhaus AD. Viruses as sole causative agents of severe acute respiratory tract infections in children. PLoS One. (2016) 11:e0150776. doi: 10.1371/journal.pone.0150776, PMID: 26964038 PMC4786225

[2] WHO. World Health Organization reveals leading causes of death and disability worldwide: 2000–2019. (2020).

[3] GBD 2017 Lower Respiratory Infections Collaborators. Quantifying risks and interventions that have affected the burden of lower respiratory infections among children younger than 5 years: an analysis for the global burden of disease study 2017. Lancet Infect Dis. (2020) 20:60–79. doi: 10.1016/S1473-3099(19)30410-4 31678026 PMC7185492

[4] Shang L Xu J Cao B. Viral pneumonia in China: from surveillance to response. Lancet Public Health. (2020) 5:e633–4. doi: 10.1016/S2468-2667(20)30264-4, PMID: 33271074 PMC7834624

[5] Zhou F Wang Y Liu Y Liu X Gu L Zhang X. Disease severity and clinical outcomes of community-acquired pneumonia caused by non-influenza respiratory viruses in adults: a multicentre prospective registry study from the CAP-China network. Eur Respir J. (2019) 54:1802406. doi: 10.1183/13993003.02406-2018, PMID: 31164430

[6] Musher DM Abers MS Bartlett JGG. Evolving understanding of the causes of pneumonia in adults, with special attention to the role of pneumococcus. Clin Infect Dis. (2017) 65:1736–44. doi: 10.1093/cid/cix549, PMID: 29028977 PMC7108120

[7] Katoh K Misawa K Kuma K Miyata T. Mafft: a novel method for rapid multiple sequence alignment based on fast fourier transform. Nucleic Acids Res. (2002) 30:3059–66. doi: 10.1093/nar/gkf436 PMID: 12136088 PMC135756

[8] Varela FH Scotta MC Polese-Bonatto M Sartor ITS Ferreira CF Fernandes IR. Absence of detection of rsv and influenza during the covid-19 pandemic in a brazilian cohort: likely role of lower transmission in the community. J Glob Health. (2021) 11:05007. doi: 10.7189/jogh.11.05007, PMID: 33791096 PMC7979153

[9] Grobben M Juncker HG van der Straten K Lavell AHA Schinkel M Buis DTP. Decreased passive immunity to respiratory viruses through human Milk during the COVID-19 pandemic. Microbiol Spectr. (2022) 10:e0040522. doi: 10.1128/spectrum.00405-22 35762813 PMC9431045

[10] Elarde J Kim JS Kavak H Züfle A Anderson T. Change of human mobility during COVID-19: a United States case study. PLoS One. (2021) 16:e0259031. doi: 10.1371/journal.pone.0259031, PMID: 34727103 PMC8562789

[11] Klee B Diexer S Horn J Langer S Wende M Ortiz D. The impact of non-pharmaceutical interventions on community non-SARS-CoV-2 respiratory infections in preschool children. BMC Pediatr. (2024) 24:231. doi: 10.1186/s12887-024-04686-2, PMID: 38561704 PMC10985994

[12] Xie JM Zhang YQ Yang K Huang XX Deng HS Wu J. Investigation of viral etiology with sever acute respiratory infection of hospitalized patients in Guangdong Province from 2019 to 2021. (in Chinese) J Pub Health Prev Med. (2023) 34:38. doi: 10.3969/j.issn.1006-2483.2023.03.008

[13] Shao LL Dong XF Ding X Jiao QJ Zhang SM Ni Q. Sepetrum analysis of acute respiratory virus infection in children in Lanzhou city from 2017 to 2018. (in Chinese) Chin Pediatr Integr Tradit West Med. (2021) 13:6. doi: 10.3969/j.issn.1674-3865.2021.03.024

[14] Yu J Li H Rao HX Lu NN Lei YJ Zhao SC. Respiratory virus detection in influenza virus-negative nasopharyngeal swabs of 150 influenza-like illness cases during flu epidemic season. (in Chinese) Shandong Med J. (2018) 58:40–43. doi: 10.3969/j.issn.1002-266X.2018.31.010

[15] Mackenzie GA Vilane A Salaudeen R Hogerwerf L van den Brink S Wijsman LA. Respiratory syncytial, parainfluenza and influenza virus infection in young children with acute lower respiratory infection in rural Gambia. Sci Rep. (2019) 9:17965. doi: 10.1038/s41598-019-54059-4, PMID: 31784567 PMC6884537

[16] Lin SX You LB Zhang YH Zhu Y Yu TT Weng YW. Investigation of pathogen spectrum of virus in acute respiratory infection in Fuzhou in 2019. (in Chinese) Chin J Exp Clin Virol. (2021) 35:188–193. doi: 10.3760/cma/j.cn112866-20200911-00240

[17] Li XQ Liu XW Zhou T Pei XF. Detection and analysis of human parainfluenza virus infection in hospitalized adults with acute respiratory tract infections. (in Chinese) J Sichuan Univ (Med Sci Edi). (2017) 48:891. doi: 10.13464/j.scuxbyxb.2017.06.019 29260527

[18] Parsons J Korsman S Smuts H Hsiao NY Valley-Omar Z Gelderbloem T. Human parainfluenza virus (HPIV) detection in hospitalized children with acute respiratory tract infection in the Western cape, South Africa during 2014-2022 reveals a shift in dominance of HPIV 3 and 4 infections. Diagnostics (Basel). (2023) 13:2576. doi: 10.3390/diagnostics13152576, PMID: 37568938 PMC10417174

[19] Lee H Kim SH Cho SJ Lee YU Lee K Lee YP. Genetic analysis of HPIV3 that emerged during the SARS-CoV-2 pandemic in Gwangju, South Korea. Viruses. (2022) 14:1446. doi: 10.3390/v14071446, PMID: 35891425 PMC9317768

[20] Zhang Y Yuan L Zhang Y Zhang X Zheng M Kyaw MH. Burden of respiratory syncytial virus infections in China: systematic review and meta-analysis. J Glob Health. (2015) 5:020417. doi: 10.7189/jogh.05.020417, PMID: 26682049 PMC4676581

[21] Falsey AR McElhaney JE Beran J van Essen GA Duval X Esen M. Respiratory syncytial virus and other respiratory viral infections in older adults with moderate to severe influenza-like illness. J Infect Dis. (2014) 209:1873–81. doi: 10.1093/infdis/jit839, PMID: 24482398 PMC4038137

[22] Price RHM Graham C Ramalingam S. Association between viral seasonality and meteorological factors. Sci Rep. (2019) 9:929. doi: 10.1038/s41598-018-37481-y, PMID: 30700747 PMC6353886

[23] Neumann G Kawaoka Y. Seasonality of influenza and other respiratory viruses. EMBO Mol Med. (2022) 14:e15352. doi: 10.15252/emmm.202115352, PMID: 35157360 PMC8988196

[24] Li Y Deng X Hu F Wang J Liu Y Huang H. Metagenomic analysis identified co-infection with human rhinovirus C and bocavirus 1 in an adult suffering from severe pneumonia. J Inf Secur. (2018) 76:311–3. doi: 10.1016/j.jinf.2017.10.012, PMID: 29111306 PMC7126302

[25] Daisley H Jr Rampersad A Daisley M Ramdin A Acco O. Coronavirus 229E with rhinovirus co-infection causing severe acute respiratory distress syndrome with thrombotic microangiopathy and death during Covid-19 pandemic: lessons to be learnt. Autops Case Rep. (2020) 10:e2020194. doi: 10.4322/acr.2020.194, PMID: 33344304 PMC7703215

[26] Amat F Plantard C Mulliez A Petit I Rochette E Verdan M. RSV-hRV co-infection is a risk factor for recurrent bronchial obstruction and early sensitization 3 years after bronchiolitis. J Med Virol. (2018) 90:867–72. doi: 10.1002/jmv.25037, PMID: 29380391 PMC7167020

[27] Regina Malveste Ito C Santos MO de Oliveira CM de Araújo KM de Souza GRL Rézio GS. Rhinovirus infection and co-infection in children with severe acute respiratory infection during the COVID-19 pandemic period. Virulence. (2024) 15:2310873. doi: 10.1080/21505594.2024.2310873, PMID: 38384141 PMC10885176

[28] Amarin JZ Potter M Thota Jm Rankin DA Probst V Haddadin Z Stewart LS. Clinical characteristics and outcomes of children with single or co-detected rhinovirus-associated acute respiratory infection in middle Tennessee. BMC Infect Dis. (2023) 23:136. doi: 10.1186/s12879-023-08084-4, PMID: 36882755 PMC9990557

[29] Cao R Du Y Tong J Xia D Song Q Xia Z. Influence of COVID-19 pandemic on the virus spectrum in children with respiratory infection in Xuzhou, China: a long-term active surveillance study from 2015 to 2021. BMC Infect Dis. (2023) 23:467. doi: 10.1186/s12879-023-08247-3, PMID: 37442963 PMC10339539

[30] Kurskaya OG Prokopyeva EA Sobolev IA Solomatina MV Saroyan TA Dubovitskiy NA. Changes in the etiology of acute respiratory infections among children in Novosibirsk, Russia, between 2019 and 2022: the impact of the SARS-CoV-2 virus. Viruses. (2023) 15:934. doi: 10.3390/v15040934, PMID: 37112913 PMC10141072

[31] Varela FH Sartor ITS Polese-Bonatto M Azevedo TR Kern LB Fazolo T. Rhinovirus as the main co-circulating virus during the COVID-19 pandemic in children. J Pediatr. (2022) 98:579–86. doi: 10.1016/j.jped.2022.03.003, PMID: 35490727 PMC9015957

[32] Teo KW Patel D Sisodia S Roland D Gaillard EA Tang JW. Rhinovirus persistence during the COVID-19 pandemic-impact on pediatric acute wheezing presentations. J Med Virol. (2022) 94:5547–52. doi: 10.1002/jmv.27986, PMID: 35811371 PMC9350342

[33] Tsang TK Du RQR Fang VJ Lau EHY Chan KH Chu DKW. Decreased risk of non-influenza respiratory infection after influenza B virus infection in children. Epidemiol Infect. (2024) 152:e60. doi: 10.1017/S0950268824000542, PMID: 38584132 PMC11062782

[34] Anestad G. Interference between outbreaks of respiratory syncytial virus and influenza virus infection. Lancet. (1982) 1:502. 10.1016/s0140-6736(82)91466-0 6121154

[35] Casalegno JS Ottmann M Duchamp MB Escuret V Billaud G Frobert E. Rhinoviruses delayed the circulation of the pandemic influenza a (H1N1) 2009 virus in France. Clin Microbiol Infect. (2010) 16:326–9. doi: 10.1111/j.1469-0691.2010.03167.x, PMID: 20121829

[36] van Asten L Bijkerk P Fanoy E van Ginkel A Suijkerbuijk A van der Hoek W. Early occurrence of influenza a epidemics coincided with changes in occurrence of other respiratory virus infections. Influenza Other Respir Viruses. (2016) 10:14–26. doi: 10.1111/irv.12348, PMID: 26369646 PMC4687500

