# Supplementary material for: Exploring the respiratory viral landscape beyond influenza and SARS-CoV-2: a 2020–2023 study in Fujian, China
Source: Front Public Health. 2025 May 14;13:1558716. doi: 10.3389/fpubh.2025.1558716 (PMC12116533; doi:10.3389/fpubh.2025.1558716)
Supplement: Supplementary file 1 [file Table_1.docx]

**Supplementary Material**

**Table S1.** Positivity rates of viral detections in 3,345 patients for ARI (From September 2020 to December 2023)

| **Pathogen** | **n(%)^a^**  **(N=3,345)** | **n (%)^b^** | | | | | ***χ*^2^ (*p*)** |
| --- | --- | --- | --- | --- | --- | --- | --- |
|  |  | 0~  (N=1,072) | 5~  (N=809) | 15~  (N=409) | 25~  (N=843) | 60~  (N=212) |  |
| Total | 1,010 (30.2) | 483(45.1) | 267(33.0) | 72 (17.6) | 145 (17.2) | 43 (20.3) | 223.533  (*p*=0.000) |
| RV | 364 (10.9) | 140 (13.1) | 101 (12.5) | 40 (9.8) | 68 (8.1) | 15 (7.1) | 17.956  (*p*=0.001) |
| HPIV | 209 (6.2) | 130 (12.1) | 40 (4.9) | 10 (2.4) | 22 (2.6) | 7 (3.3) | 98.566  (*p*=0.000) |
| HRSV | 152 (4.5) | 95 (8.9) | 27 (3.3) | 5 (1.2) | 12 (1.4) | 13 (6.1) | 79.353  (*p*=0.000) |
| HAdV | 144 (4.3) | 68 (6.3) | 54 (6.7) | 5 (1.2) | 16 (1.9) | 1 (0.5) | 50.692  (*p*=0.000) |
| HMPV | 103 (3.1) | 48 (4.5) | 35 (4.3) | 4 (1.0) | 14 (1.7) | 2 (0.9) | 26.215  (*p*=0.000) |
| HCoV | 103 (3.1) | 41 (3.8) | 26 (3.2) | 11 (2.7) | 19 (2.3) | 6 (2.8) | 7.339  (*p*=0.501) |
| HBoV | 36 (1.1) | 25 (2.3) | 6 (0.7) | 1 (0.2) | 2 (0.2) | 2 (0.9) | 50.692  (*p*=0.000) |

^a^(%) are calculated on total number of samples. ^b^(%) are calculated on number of samples within each respective age group.
